# The Effect of Congenital and Acquired Bilateral Anophthalmia on Brain Structure

**DOI:** 10.1080/01658107.2020.1856143

**Published:** 2021-03-01

**Authors:** Holly Bridge, Gaelle S. L. Coullon, Rupal Morjaria, Rebecca Trossman, Catherine E Warnaby, Brian Leatherbarrow, Russell G. Foster, Susan M. Downes

**Affiliations:** a Wellcome Centre for Integrative Neuroimaging, Nuffield Department of Clinical Neurosciences, University of Oxford, John Radcliffe Hospital, Oxford, UK; b Oxford Eye Hospital, John Radcliffe Hospital, Oxford, UK; c Birmingham Midland Eye Centre, Sandwell & West Birmingham Hospitals NHS Trust, Birmingham, West Midlands, UK; d Manchester Royal Eye Hospital NHS Trust, Manchester, UK; e Nuffield Department of Clinical Neurosciences, Sleep & Circadian Neuroscience Institute (SCNi) and Nuffield Laboratory of Ophthalmology, Oxford, UK

**Keywords:** Anophthalmia, magnetic resonance imaging, diffusion imaging, optic nerve, cortical thickness, grey matter

## Abstract

The aim of this study was to compare the pattern of changes in brain structure resulting from congenital and acquired bilateral anophthalmia. Brain structure was investigated using 3T magnetic resonance imaging (MRI) in Oxford (congenital) or Manchester (acquired). T1-weighted structural and diffusion-weighted scans were acquired from people with anophthalmia and sighted control participants. Differences in grey matter between the groups were quantified using voxel-based morphometry and differences in white matter microstructure using tract-based spatial statistics. Quantification of optic nerve volume and cortical thickness in visual regions was also performed in all groups. The optic nerve was reduced in volume in both anophthalmic populations, but to a greater extent in the congenital group and anophthalmia acquired at an early age. A similar pattern was found for the white matter microstructure throughout the occipitotemporal regions of the brain, suggesting a greater reduction of integrity with increasing duration of anophthalmia. In contrast, grey matter volume changes differed between the two groups, with the acquired anophthalmia group showing a decrease in the calcarine sulcus, corresponding to the area that would have been peripheral primary visual cortex. In contrast, the acquired anophthalmia group showed a decrease in grey matter volume in the calcarine sulcus corresponding to the area that would have been peripheral primary visual cortex. There are both qualitative and quantitative differences in structural brain changes in congenital and acquired anophthalmia, indicating differential effects of development and degeneration.

## Introduction

A large proportion of the human brain is dedicated to the processing of visual information and in the case of sensory loss, this neural tissue may be used for other functions. In many cases, however, the visual loss is not complete, leaving some residual visual perception, even if only the ability to perceive light. The most extreme form of visual deprivation occurs when there is no input from the eyes due to their absence, known as anophthalmia.

Congenital bilateral anophthalmia is the absence of both eyes and is characterised by a ‘visual’ system that has never experienced pre- or post-natal visual stimulation. Congenital anophthalmia without any other neurological impairment provides an ideal population to study the effects of complete visual deprivation on healthy brain tissue. The same is true of acquired bilateral anophthalmia where both eyes are lost either through trauma or surgical removal of both eyes due to diseases such as retinoblastoma, or end-stage glaucoma, and thus occurs after visual system development. Comparing congenital and acquired bilateral anophthalmia allows the study of visual pathway and whole-brain reorganisation and/or degeneration in the context of complete vision loss taking place either before (congenital) or after (acquired) visual system development.

Structural brain changes due to both congenital and acquired blindness have been described in grey and white matter. Grey matter atrophy (up to 20–25%) has been noted in primary ‘visual’ areas^1,2^ and extra-striate regions^2^ in early blind individuals compared with sighted controls. Reduced white matter volume and fractional anisotropy (FA) were found in the optic chiasm, optic nerves, and optic radiations, as well as regions of the occipital lobe and corpus callosum^1,2^ in congenital and early-onset blindness, although the global organisation of the splenium remains unchanged.^3^ While there is considerable variability in the patterns of structural brain changes in blindness, some features are consistently present. This has been investigated in a large group (n > 50) of blind participants with different underlying causes, who showed consistent reduction in lateral geniculate nucleus (LGN) and V1 volume and increase in V1 cortical thickness. Furthermore, white matter microstructure within the optic radiation also showed abnormalities across the blind participants.^4^ However, although these differences are consistent across congenital and acquired blindness, they are likely to result from different plastic processes; altered development in the former case and degeneration in the latter.

Here we compare individuals with congenital and acquired bilateral anophthalmia to characterise the patterns of degeneration and reorganisation in these two different groups. Specifically, the anterior visual pathway (optic nerve) and cerebral cortex grey and white matter are quantified relative to healthy, sighted control participants.

## Materials and methods

### Participants

Fifteen participants with anophthalmia and 20 control participants were recruited in total. Of the participants with anophthalmia, six had congenital bilateral anophthalmia (mean age 29 years, range 21–37 years, four males, two females), one had congenital bilateral microphthalmia and eight had acquired bilateral anophthalmia (mean age 51 years, range 25–70 years, three males; five females) (see Table 1 for details). Of the congenital anophthalmic group: case 1 had congenital anophthalmia with apathogenic variant in the gene OTX2; case 6 had congenital microphthalmia due to Norrie disease with a pathogenic variant in the NDP gene. Neither of these cases, nor any of the others had any syndromal, in particular neurological, symptoms, or signs. Twenty sighted controls with normal vision (Snellen best corrected visual acuity of 6/6 or better) were also recruited. Of these, 12 were controls for the congenital anophthalmia group (mean age 31 years, range 24–46, six males, six females) and 8 were controls for the acquired anophthalmia group (mean age 41 years, range 33–61 years, two males, six females). Due to the location of the participants, scanning was carried out at two sites: participants with congenital anophthalmia and their sighted controls at the University of Oxford (Oxford site); and acquired anophthalmia participants and their controls at the University of Manchester (Manchester site). This study was granted ethical approval by the South Central Oxford Research Ethics Committee (B/11/SC/0093) and all participants gave written informed consent prior to participation.

**Table 1.** Demographics and blindness information for all anophthalmic cases

Subject ID	Gender	Age (years)	Age blindness onset (years)	# years blind	% of life spent blind
Congenital 1	Male	34	0	34	100%
Congenital 2	Female	38	0	38	100%
Congenital 3	Female	23	0	23	100%
Congenital 4	Male	29	0	29	100%
Congenital 5	Male	30	0	30	100%
Congenital 6	Male	21	0	21	100%
Acquired 1	Male	54	2	52	96%
Acquired 2	Male	47	1	46	98%
Acquired 3	Male	61	36	25	41%
Acquired 4	Female	60	40	20	33%
Acquired 5	Female	70	56	14	20%
Acquired 6	Female	25	20	5	20%
Acquired 7	Female	28	21	7	25%
Acquired 8	Female	65	35	30	46%

### MR imaging acquisition

#### Oxford

Images were acquired using a Siemens Verio 3-Tesla whole body magnetic resonance imaging (MRI) scanner and a 32-channel coil at the Functional Magnetic Resonance Imaging of the Brain Centre (University of Oxford). Structural images were acquired at 1 mm isotropic resolution using a T1-weighted magnetization prepared rapid gradient echo (MPRAGE) sequence (TR = 2040 ms, TE = 4.7 ms, flip angle = 8°, 192 transverse slices, 1 mm isotropic voxels). Diffusion-weighted images were acquired axially using echo-planar imaging with 2 mm^3^ isotropic voxels. The diffusion weighting was isotropically distributed through space along 60 directions using a b-value of 1500 s/mm^2^. Four volumes with no diffusion weighting were acquired during each sequence.

#### Manchester

Images were acquired using a Philips 3-Tesla whole body MRI scanner and a 32-channel coil at the NIHR/Wellcome Trust Clinical Research Facility (University of Manchester). Structural images were acquired at 1 mm isotropic resolution using a T1-weighted MPRAGE sequence (TR = 2439 ms, TE = 8.7 ms, flip angle = 8°, 192 transverse slices, 1 mm isotropic voxels). Diffusion-weighted images were acquired axially using echo-planar imaging with 2 mm^3^ isotropic voxels. The diffusion weighting was isotropically distributed through space along 61 directions using a b-value of 1500 s/mm^2^. One volume with no diffusion weighting was acquired during each sequence.

### Data analysis

Structural MRI data were analysed using voxel-based morphometry (VBM) in order to investigate voxel-wise differences in local grey matter volume between groups, specifically FSL-VBM (http://fsl.fmrib.ox.ac.uk/fsl/fslwiki/FSLVBM), an optimised VBM protocol^5^ carried out with FSL tools.^6^ Images from each site (Oxford and Manchester) were analysed separately. First, structural images were brain-extracted, tissue-type segmented and registered to MNI-152 standard space using non-linear registration.^7^ The resulting images were averaged and flipped along the x-axis to create a left-right symmetric, study-specific grey matter template. Second, all native grey matter images were non-linearly re-registered to this study-specific template and “modulated” to correct for local expansion (or contraction) due to the non-linear component of the spatial transformation. The modulated grey matter images were then smoothed with an isotropic Gaussian kernel (3 mm sigma).

Diffusion data were analysed by calculating FA maps and comparing local FA between blind and sighted groups. FA is a measure of diffusion direction strength and can be used as an indication of fibre density. Voxel-wise statistical analysis of the FA data was carried out using TBSS (Tract-Based Spatial Statistics,^8^ part of FSL).^6^ Images from each site (Oxford and Manchester) were analysed separately. First, FA images were created by fitting a tensor model to the raw diffusion data using FDT and then brain-extracting each image. All subjects’ FA data were then aligned to MNI-152 standard space using the non-linear registration.^7^ Next, the mean FA image was created and thinned to create a mean FA skeleton that represents the centres of all tracts common to the group. Each subject’s aligned FA data were then projected onto this skeleton (shown in green in figures) and the resulting data fed into voxel-wise cross-subject statistics. A similar procedure was undertaken to quantify differences in mean diffusivity (MD). Data from one participant with congenital anophthalmia and one with acquired anophthalmia had to be discarded due to movement artefacts.

For both grey matter volume and FA, voxel-wise general linear modelling (GLM) was applied using permutation-based non-parametric testing (5000 permutations) to investigate significant differences between the ‘anophthalmic’ and control groups with threshold-free cluster enhancement correction. However, as the acquired anophthalmic and Manchester controls were not well age matched, age was added as a covariate of no interest in both VBM and TBSS design matrices. An additional VBM and TBSS design was used to investigate the negative effect of blindness years in the Manchester data; demeaned percentage of total lifetime spent blind was calculated for each subject (0% for Manchester controls, see Table 1 for acquired anophthalmics). The Juelich Histological Atlas, Harvard-Oxford Cortical Structural Atlas, and JUH White-Matter Tractography Atlas as implemented in fslview (version 3.2.0) were used to identify structures and regions of interest for further analyses.

#### Optic nerve analysis

Structural T1-weighted MPRAGE images were used to assess the integrity of the optic nerve in congenital and acquired anophthalmia. In order to quantitatively compare optic nerve integrity across subject groups, an approximate left and right optic nerve mask was drawn coronally for a maximum of 15 slices within the orbit until the orbito-canalicular junction (range 12–15 slices depending on visibility), starting at the first slice posterior to the globe when the optic nerve could be identified as a distinct structure. The masks were drawn larger than the actual optic nerve, as the size of the mask was then limited by the intensity of the image, to ensure that only white matter voxels were included. The same threshold value was used across sighted and anophthalmic participants from a particular site (Oxford and Manchester), and the thresholds were chosen to maximise the optic nerve size in control participants, which ensuring volume at the two sites was as comparable as possible. The difference in scanner and scan sequence at the two scan sites, particularly the presence and absence of fat suppression meant that different intensity values were required. The approach is similar to the method used previously to quantify the optic tract in patients with hemianopia,^9^ and is designed to ensure the definitions are as objective and replicable as possible. This is important because although the analysis was performed without researcher knowledge of participant identity, the usual types of blinding that would be used for the brain, such as removing non-brain tissue were not possible. The volume (mm^3^) of these masks was extracted, summed across the left and right nerves and mean volume for sighted and anophthalmic groups were compared separately for the two sites.

#### Cortical thickness

Structural T1-weighted MPRAGE images were also analysed using Freesurfer (http://surfer.nmr.mgh.harvard.edu).^10^ The images were segmented into tissue types^11^ and average cortical thickness values were calculated from those areas that can be reliably defined based on anatomical features (V1, V2, MT/V5, primary somatosensory cortex: Brodmann areas 3a and 3b and primary motor cortex: Brodmann areas 4a and 4p).

In addition to standard t-tests that were performed on the samples to determine whether there was a significant difference between the anophthalmic and control groups, a Bayesian t-test was performed using the open-source software package JASP.^12^ Bayesian analyses permit a test of the relative strength of evidence for the null hypothesis (H_0_: no difference in cortical thickness) versus the alternative hypothesis (H_1_: difference in cortical thickness between the two groups).^13^

## Results

### The absence of the eyes impacts upon the optic nerve

**Figure 1.**
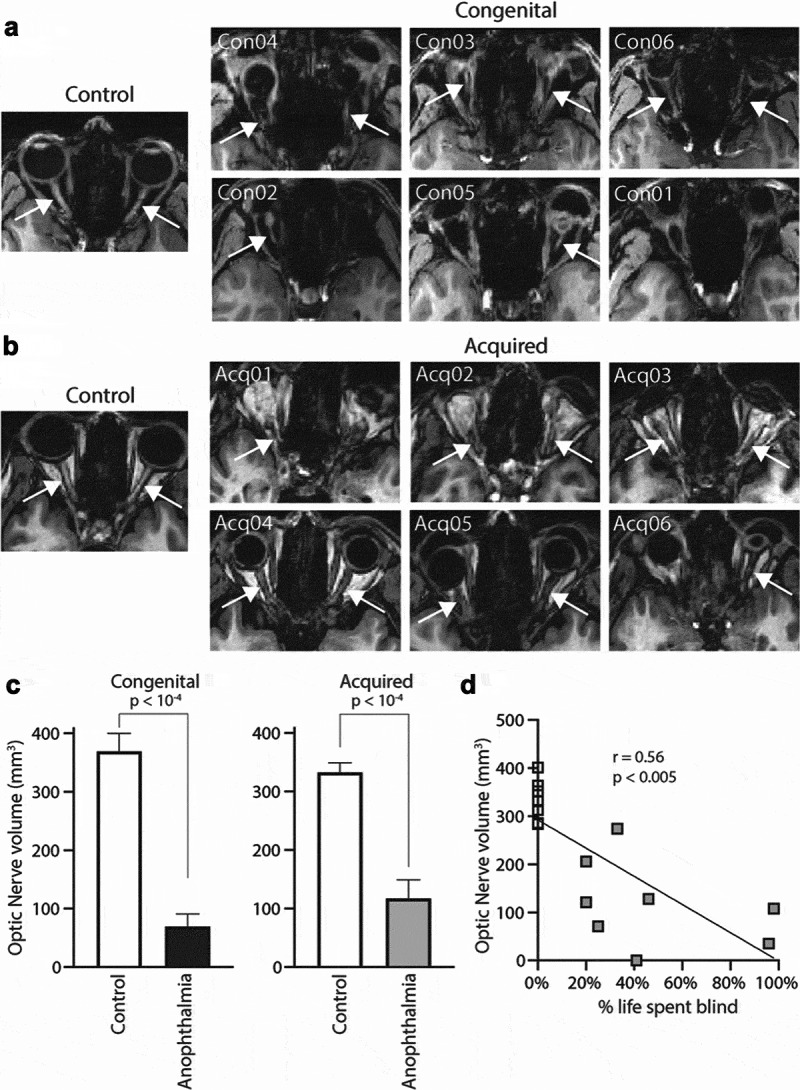
Optic nerve analysis using T1-weighted MPRAGE images. Axial slices show the expected location of the optic nerve in all congenital (a) and six of the acquired (b) anophthalmic cases. For comparison, the optic nerves from two example controls are also shown. In each case, white arrows point to the optic nerve if present. Mean optic nerve volume for each group (c) is plotted. Error bars represent standard error of the mean. Finally, optic nerve volume for each acquired anophthalmic case and relevant controls was plotted against % life spent blind, revealing a significant negative correlation (d, Pearson’s r = 0.56, *p* < .005)

Structural T1-weighted MPRAGE images were used to assess the integrity of the optic nerve in congenital and acquired anophthalmia. Figure 1 shows axial slices for all congenital (A) and six of the acquired (B) anophthalmic cases where the optic nerve is normally located. For comparison, a sighted subject from each site is also shown. Where identified, the optic nerve is indicated by a white arrow. The optic nerve was significantly reduced in both anophthalmia groups, while the congenital group appeared to show even greater reduction than the acquired group (C). Optic nerve volume was significantly lower in both blind groups compared to their respective control groups (independent-samples t-test; congenital group t = 6.5; d.f. = 17; *p* < 1x10^−4^; acquired group t = 5.8; d.f. = 16; *p* < 1x10^−4^). Optic nerve volume for each individual subject at the Manchester site is plotted against percentage of life spent blind in D. There is a significant negative correlation across both sighted and anophthalmic cases (Pearson’s R = 0.56, *p* < .005).

### Grey matter volume reduction present in acquired anophthalmia

**Figure 2.**
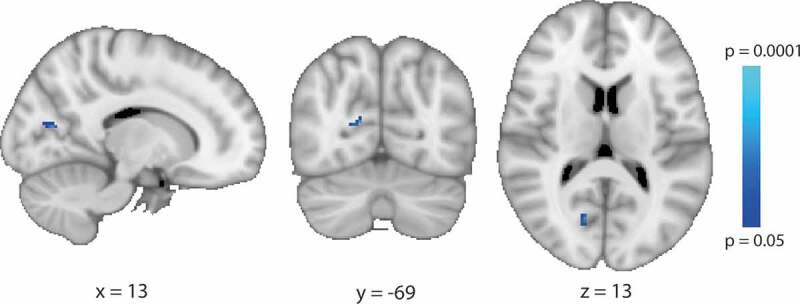
Reduced grey matter volume in acquired anophthalmia compared to sighted controls in the anterior calcarine in the right hemisphere. The colour represents the significance of the difference between the two groups. Data are shown on the MNI standard brain

There were no brain areas in either the congenital or acquired anophthalmia groups that showed increased grey matter volume compared with the sighted control group using a whole brain approach. In contrast, the group with acquired anophthalmia showed a decrease in grey matter volume in the anterior calcarine region compared with sighted controls (Figure 2; shown on MNI standard brain). This region corresponds to primary ‘visual’ cortex (Juelich Histological Atlas). No significant differences were found in the congenitally anophthalmic group, although it should be noted that the sample sizes are relatively small.

Next, we investigated whether any changes in grey matter volume in acquired anophthalmia (increases or decreases compared with sighted controls) were related to age of blindness onset. This was done with an additional VBM group comparison looking at negative effects of blindness years (measured as demeaned percentage of total lifetime spent blind, in order to account for age differences between the groups). This yielded no significant results at a group level. In order to look at both blind and sighted groups, grey matter volume in ‘visual’ areas V1, V2 and V5/MT was extracted in all subjects (Juelich Histological Atlas definitions; V1 and V2 thresholded at 40%, MT/V5 thresholded at 15%). No significant correlations were found between percentage lifetime spent blind and grey matter volume in any regions (left and right hemisphere together or separately). Additional comparisons were made with age of blindness onset and number of years of blindness, which also yielded no significant correlations.

### Increased V1 cortical thickness in congenital, but not acquired anophthalmia

**Figure 3.**
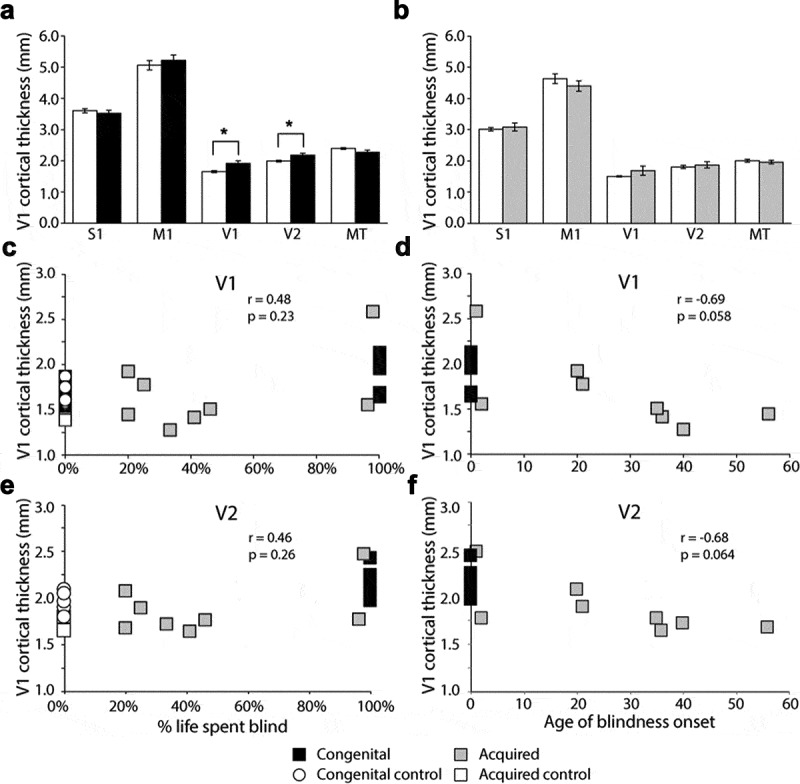
Cortical thickness in visual regions of interest. A shows the cortical thickness measures in the congenital anophthalmia group and controls. Cortical thickness in V1 and V2 of the congenital anophthalmia groups was significantly greater than sighted controls. B shows the same data for the acquired anophthalmia group. There are no significant differences in cortical thickness in any areas in the acquired anophthalmia group. C shows the correlation between % life spent blind and V1 cortical thickness. D shows the correlation between age of blindness onset and V1 cortical thickness. Asterisks indicate *p* < .005

Cortical thickness in primary visual cortex (V1) has consistently been shown to be greater in congenital blindness compared with sighted controls. In the congenital anophthalmia group, regions corresponding to both V1 (t = 3.7; d.f. = 17; *p* < .005) and V2 (t = 3.3; d.f. = 17; *p* < .005) were thicker than in the sighted control group. This was not the case for any of the other regions shown in Figure 3a. Furthermore, there were no significant differences between the acquired anophthalmia group and the relevant sighted controls (Figure 3b). To aid interpretation of the null effect in the acquired group, a Bayesian t-test was also performed on these data. The Bayes factor (BF_01_) for the null hypothesis (i.e. no difference in cortical thickness) was 1.4 times more likely that the alternative hypothesis (a difference in cortical thickness) in V1 and 2.1 times more likely in V2. This suggests the lack of difference is not due to insufficient power.

Given the wide range in the duration of anophthalmia in this group, a second analysis was undertaken in which cortical thickness in V1 and V2 was correlated with both the age of blindness onset and the proportion of life spent blind. Using only the acquired anophthalmia group there was no correlation with per cent life spent blind in either V1 (r = 0.48; *p* = .23; Figure 3c) or V2 (r = 0.46; *p* = .26; Figure 3e). The age of onset of blindness had a stronger relationship with cortical thickness, but was not significant in either area, particularly when Bonferroni correction was applied (V1: r = −0.69; *p* = .058; Figure 3d V2: r = −0.68; *p* = .064; Figure 3f). A Bayesian correlation was also performed on these data. In contrast to the result in the previous section, the Bayes factor (BF_01_) was below 1, indicating little evidence for the null hypothesis (no relationship between cortical thickness and age of blindness onset: BF_01_ for V1 = 0.49; BF_01_ for V2 = 0.53). Thus, it is possible there may be a correlation, but we do not have sufficient power to detect it.

A potentially interesting result is the discrepancy between the two individuals with acquired anophthalmia who have spent over 90% of their lives blind. One shows increased cortical thickness whereas the other is comparable to control participants. Both are the same gender and fall within a similar age range at time of scan (45–55 years). However, inspection of the optic nerve volume indicates that the individual with thicker cortex also shows greater optic nerve integrity, suggesting perhaps that relative preservation of tissue in the anterior visual system can lead to increased tissue downstream. Alternatively, it may be that the participant with increased cortical thickness has greater cross-modal plasticity, comparable to the congenital anophthalmia group. Whether the small difference in age of blindness onset (one year compared to two years) could account for this difference remains to be determined in larger studies.

### White matter microstructure altered in both congenital and acquired anophthalmia

**Figure 4.**
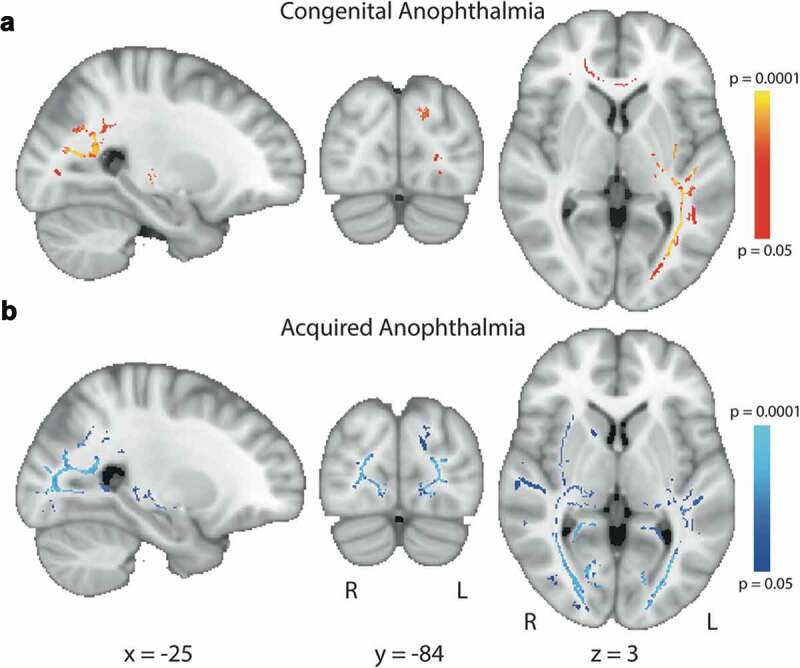
Reduced FA in congenital (a) and acquired (b) anophthalmia groups compared to sighted controls. Red-yellow regions indicate significantly lower FA in the congenital anophthalmia group and blue shows those significantly lower in the acquired anophthalmia group. For visualisation purposes, all statistical maps are thresholded at *p* < .05 (after TFCE correction for multiple comparisons)

There were no significant increases in FA in either the congenital or acquired anophthalmia groups compared to the sighted control groups. Figure 4 shows the regions of white matter showing reduced FA in the congenital (A) and acquired (B) anophthalmia groups compared to the relevant controls. Results are thresholded at *p* < .05 corrected using Threshold Free Cluster Enhancement (TFCE).

FA was significantly reduced bilaterally along the optic radiations in both congenital and acquired anophthalmia, although this reduction appears primarily in the left optic radiations in the congenital group. In the acquired group, FA reductions in the optic radiations overlapped with and extended to the longitudinal fasciculus (inferior and superior) and fronto-occipital fasciculus (according to JUH White-Matter Tractography atlas; MNI coordinates X = +25 to +35, Y = −31 to +19, Z = −14 to +26).

In the acquired anophthalmia group only (Figure 4b), FA was also reduced in the thalamus (MNI coordinates −22, −30, +39). This region near the end of the anterior thalamic radiations is known to project to the occipital, posterior-parietal and temporal cortices (according to the Oxford Thalamic Connectivity Probability atlas) and corresponds to where the pulvinar is normally located (Leh et al., 2008). Finally, FA in the congenital group only (Figure 4a) was reduced in the anterior corpus callosum (see Figure 4a at MNI coordinate Z = +2).

To confirm that different FA results in congenital anophthalmia versus acquired groups were not due to different group sizes (five congenital cases as opposed to seven acquired cases), an additional TBSS analysis was performed for the Manchester data with only the five youngest acquired anophthalmic cases (to match the group size and age of the congenital cases). Reduced FA (compared to sighted controls) in this smaller acquired group did not differ from the larger group.

**Figure 5.**
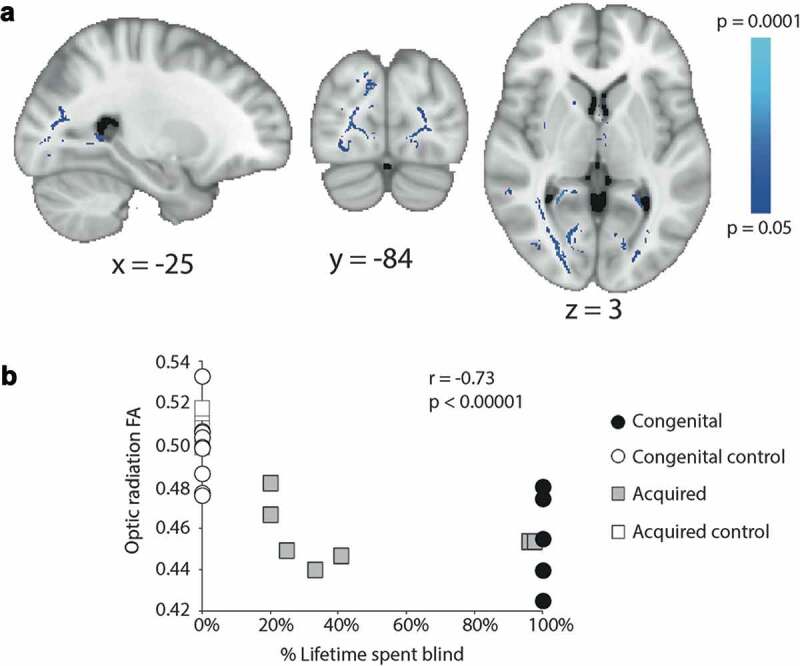
A shows the white matter in which the reduction in FA correlates inversely with the percentage of life spent blind across the acquired anophthalmia group. Mean optic radiation FA (both hemispheres) plotted against percentage of lifetime spent blind for the congenital anophthalmics (black circles, all 100%), acquired anophthalmics (grey squares), congenital controls (white circles, all 0%) and acquired controls (white squares, all 0%)

A second analysis was performed on the acquired anophthalmia group to determine the regions of white matter that exhibited a change in FA related to the percentage of life spent blind. Figure 5a shows the white matter tracts in the brain that showed an inverse relationship with the percentage of life spent blind, that is that the longer the period spent blind, the lower the FA. This correlation was restricted to the optic radiation. Indeed, when the FA extracted from the entire optic radiation was correlated with percentage of life spent blind across all participants (Figure 5b) there was a significant negative correlation (Pearson’s r = −0.73, *p* < .00001). However, there was no significant correlation in the blind groups alone, so this result is likely driven by overall lower FA in both blind groups compared to sighted controls.

## Discussion

The current study has considered the most extreme version of blindness, bilateral anophthalmia, in which the eyes are absent. Two separate populations were considered, a congenital group in whom the eyes did not develop, and an acquired one in whom the eyes were removed after birth. While the resulting absence of vision is the same in both populations, the visual and developmental experience is considerably different, reflected in some differences between groups.

### The anterior visual system is significantly altered in both types of anophthalmia

The considerable changes in the anterior visual system, previously shown in the congenital group using detailed MRI of the cranial nerves,^14^ are not surprising when taking into consideration difference in time of onset of blindness. In congenital cases, the absence of the globe will invariably affect development of the optic nerve, leading to the total absence or extreme hypoplasia. Where the globes have been removed, as in the case of acquired anophthalmia, the previously intact optic nerves become deafferented, and the ganglion cells will undergo anterograde (Wallerian) degeneration. That this is a progressive degeneration is reflected in the correlation between optic nerve and the age of blindness onset. While the correlation is significant, there is also a reduction in optic nerve volume in those who had their eyes removed in the sixth or seventh decade. This is likely to reflect, in part, the age-related decrease in retinal ganglion fibres noted previously.^15^ It is perhaps surprising how small the reduction of optic nerve volume is in the participants whose eyes were removed in adulthood. Given the direct damage to the cell bodies and consequent loss of activity, greater volume loss might be expected.

### Grey matter changes differ in acquired and congenital anophthalmia

There were two main results from the analysis of grey matter in these anophthalmic populations, both relating to the calcarine sulcus, the cortical region that would be classified as V1 in sighted participants. Firstly, the acquired anophthalmia population showed a significant decrease in grey matter in the anterior portion of this structure. This decrease is likely the result of trans-synaptic degeneration resulting from the removal of the eyes. Since people who become blind later in life tend to show less reorganisation^16–20^ than those who are congenitally blind, the reduction in input from the eyes is likely to be the main driver of structural change. In congenitally anophthalmic participants cross-modal plasticity ensures that the occipital lobe, including the calcarine sulcus, continues to receive both cortico-cortical input and some subcortical input.^19,21–32^ In addition to the functional activation, the significant increase in cortical thickness in this region that was present for the congenitally anophthalmic group is likely to counteract any decrease in volume. Increased thickness in the calcarine sulcus has been found consistently across studies of congenitally blind people,^4,33–37^ and appears to reflect a lack of the usual pruning that makes V1 one of the thinnest regions of the cortical ribbon. A large study of people with blindness acquired at different ages also appears to support this finding, indicating that V1 becomes thinner during childhood and adolescence.^38^ Thus, as found here, people in whom blindness occurs after this period do not show increased cortical thickness in V1. An alternative explanation, however, using multi-modal MRI has shown that apparent thinning in extra-striate cortex may actually be an increase in cortical myelination, which affects the definition of the grey and white matter boundary.^39^ Whether this is also the case for V1 remains to be determined.

### Consistent white matter changes in optic radiation in both congenital and acquired anophthalmia

As in the majority of previous studies of blind populations,^37,40–44^ changes in the white matter microstructure of the optic radiations represent the most prominent difference between both anophthalmia groups and sighted controls. This consistency has been shown previously by Reislev et al.^42^ for congenital and late acquired blindness. Since much of the optic radiation shows a significant inverse correlation with the proportion of life spent blind in the acquired anophthalmia group, it is likely that the change is due to degeneration of these deafferented fibre bundles. This finding is consistent with that of Reislev et al. in acquired blindness, due to heterogeneous causes, although there was no such correlation in the study of Wang et al.^44^

The reduction in FA in the optic radiations seen in the congenitally anophthalmic group presumably reflects the reduction in usage of this normally dominant tract. The atrophy of the LGN in this population^37^ and other congenitally blind populations^4,45^ is consistent with a loss of major input along the optic radiations. Instead the input to the occipital lobe is projected predominantly via cortico-cortical connections. The reduction of FA in the acquired group appears to be more extensive than the congenital group, a finding that is robust even when subject numbers are comparable. While this may seem counterintuitive, anophthalmia that is acquired later in life will not lead to extensive cross-modal plasticity,^17^ and therefore visual pathways will no longer be used and will likely atrophy. In contrast, the extensive cross-modal plasticity is present in congenital anophthalmia^21–23^ is likely to protect pathways beyond the optic radiations.

## Conclusion

Bilateral anophthalmia is the most extreme version of blindness, and this is reflected in the extensive atrophy of the optic nerve and abnormalities of the white matter tracts within the occipital lobe.
